# Nocturnal hypoxia in patients with sleep disorders: exploring its role as a mediator between neurotic personality traits and psychological symptoms

**DOI:** 10.3389/fpsyt.2024.1442826

**Published:** 2024-12-06

**Authors:** Fei Jiang, Jinsong Huang, Lijun Fan, Xiaoyan Dong, Chunyan Yang, Wenzhu Zhou

**Affiliations:** ^1^ Department of Psychiatry, Dalian Seventh People’s Hospital, Dalian, China; ^2^ Department of Psychiatry, The First Affiliated Hospital of China Medical University, Shenyang, China

**Keywords:** nocturnal hypoxia, mediation analysis, neuroticism, psychological symptoms, sleep disorders

## Abstract

**Introduction:**

Sleep disorders often coexist with personality and psychological issues, alongside nocturnal hypoxia. This study investigates the potential mediating role of nocturnal hypoxia between personality traits and psychological symptoms in individuals with sleep disorders.

**Methods:**

A cohort comprising 171 participants reporting sleep disturbances was recruited from Dalian Seventh People’s Hospital. Psychological symptoms were assessed using the Symptom Checklist-90-R (SCL-90-R), while personality traits were evaluated using the Eysenck Personality Questionnaire (EPQ). Nocturnal hypoxia status was determined through overnight polysomnography.

**Results:**

Mediation analysis, conducted using SPSS 23.0, demonstrated that the cumulative time of nocturnal peripheral oxygen saturation (SpO2) < 85% (T85) partially mediated the relationship between neuroticism and various psychological symptoms, including somatization (c=0.207, c’=0.164, a*b=0.043, proportion of mediation 20.8%), interpersonal sensitivity (c=0.360, c’=0.326, a*b=0.034, proportion of mediation 9.6%), depression (c=0.277, c’=0.234, a*b=0.042, proportion of mediation 15.3%), anxiety (c=0.240, c’=0.199, a*b=0.041, proportion of mediation 16.9%), hostility (c=0.241, c’=0.205, a*b=0.036, proportion of mediation 14.9%), phobic anxiety (c=0.271, c’=0.241, a*b=0.030, proportion of mediation 11.1%), and psychoticism (c=0.298, c’=0.266, a*b=0.032, proportion of mediation 10.8%).

**Discussion:**

These findings underscore the potential mediating role of nocturnal hypoxia in the association between neuroticism personality traits and psychological symptoms among individuals with sleep disorders. Our research holds considerable significance in advancing the quest for personalized treatments targeting psychological symptoms in individuals with sleep disorders.

## 1 Introduction

Sleep disorders often coexist with personality and psychological issues, alongside nocturnal hypoxia. Personality traits and psychological symptoms are fundamental aspects of human behavior and mental well-being (1). Personality traits encompass enduring patterns of cognition, emotion, and behavior that consistently characterize individuals across diverse contexts. In contrast, psychological symptoms represent deviations from typical functioning and encompass a broad spectrum of psychological phenomena, including mood dysregulation, cognitive disturbances, and behavioral aberrations. Extensive research has investigated the associations between personality traits and various psychological disorders, including depression, anxiety, and schizophrenia (2–4). For example, individuals with elevated levels of psychopathic traits often exhibit deficits in empathy and engage in coercive behaviors, with a propensity towards multiple forms of violence (5). Specific personality traits may predispose individuals to particular psychological symptoms, modulate symptom severity, and influence treatment outcomes. Maladaptive personality traits characterized by internalizing tendencies and psychoticism have been linked to heightened psychological distress (6). Additionally, vulnerability traits have been reported to negatively impact anxiety and depression, especially in the context of the COVID-19 pandemic (7). While personality traits generally demonstrate relative stability over time, psychological symptoms exhibit variability in intensity and manifestation, often fluctuating in response to internal and external stressors. Moreover, personality traits are intricately intertwined with stress responsivity under certain psychological circumstances. For instance, research has shown that levels of salivary enzymes may increase in individuals exhibiting traits such as neuroticism, extraversion, agreeableness, and rumination in response to psychological stressors (8). Studies have also explored the relationship between personality traits and specific psychological symptoms. For instance, research on internet addiction in teenagers found that certain personality traits were associated with higher scores on measures of psychological distress (9). Similarly, studies examining the Big Five personality traits revealed positive associations between neuroticism and openness and COVID-19 anxiety (10). Additionally, self-reported personality, particularly neuroticism, has been prospectively associated with a broad spectrum of behavioral symptoms among individuals with cognitive impairment nearing the end of life (11). Furthermore, alterations in personality traits such as neuroticism, extraversion, openness to experiences, and conscientiousness have been linked to symptoms of apathy and affective symptoms among patients diagnosed with mild cognitive impairment (12). Furthermore, alterations in personality traits such as neuroticism, extraversion, openness to experiences, and conscientiousness have been linked to symptoms of apathy and affective symptoms among patients diagnosed with mild cognitive impairment (13). In the realm of psychotherapy, a cohort study discovered that individuals with a combined high extraversion and high neuroticism personality score at baseline were more likely to receive benzodiazepine prescriptions (14).

On the other hand, Hypoxia has been identified as a factor associated with psychological symptoms. Previous research has indicated that prolonged exposure to anoxic environments can precipitate neuropsychiatric disorders and heighten the vulnerability to depression, anxiety, and other psychological issues (15, 16). Furthermore, residing at high altitudes has been linked to an increased risk of depression and suicide (17, 18). Acute exposure to high-altitude hypoxia has been shown to induce adverse neurological effects (19). Moreover, emerging lines of evidence suggest a potential role of hypoxia in the pathophysiology of schizophrenia (20–23). Intriguingly, depression and anxiety disorders are frequently observed as comorbidities in individuals with hypoxia associated with chronic obstructive pulmonary disease (COPD) (24). Obstructive sleep apnea (OSA), characterized by recurrent episodes of intermittent hypoxia during sleep, is prevalent in approximately 11%–18% of individuals diagnosed with major depressive disorder (25). Indeed, various researchers have delineated differing patterns of effect resulting from hypoxic events on the brain’s neuropathological and cognitive processes. Some describe a diffuse pattern, wherein there is a global deterioration across various brain regions and functions (26). This diffuse effect suggests widespread damage throughout the brain. In contrast, others propose that certain brain regions are affected at different rates, with specific cortical regions known as ‘watershed’ areas being particularly vulnerable (27). These watershed regions rely heavily on cerebral arteries for an efficient and continuous vascular supply. Consequently, they are more susceptible to damage during hypoxic events. Additionally, studies have identified specific brain regions within the temporal lobes, including the hippocampus, as being especially prone to the effects of hypoxia (28–30). These regions are noted for their high metabolic activity and are therefore more sensitive to disruptions in oxygen supply. Damage to the hippocampus, for example, can have profound implications for memory formation and cognitive function. Indeed, some researchers have proposed that the structural and pathophysiological consequences following hypoxia are primarily dependent on the nature and severity of the injury (31). This suggests that the extent of damage and the specific mechanisms of injury play crucial roles in determining the outcomes of hypoxic brain injury. A literature review has indicated that the total duration of unconsciousness following a hypoxic brain injury is closely associated with long-term cognitive prognosis. This suggests that the duration of impaired consciousness serves as an important predictor of cognitive outcomes, with longer periods of unconsciousness generally correlating with poorer cognitive prognosis. Furthermore, scholars have argued that the extent of impairment is directly linked to the site and brain structures affected by the hypoxic event (27). In other words, the specific brain regions and structures that sustain damage during a hypoxic event play a critical role in determining the severity and nature of cognitive deficits experienced by individuals following such an injury.

Clinical studies have shown that individuals with neurotic personality traits have an increased susceptibility to respiratory diseases primarily characterized by hypoxia. A cohort study of older adults found that individuals with high levels of neuroticism had lower peak expiratory flow and were more likely to develop COPD and experience dyspnea (32). In patients with stable COPD, dyspnea scores were correlated with indices of anxiety, depression, and neuroticism (33). Additionally, research indicates that neuroticism, agreeableness, and conscientiousness personality traits significantly influence COPD Assessment Test scores (34). Moreover, the diagnosis of asthma and poor asthma control in adolescents is associated with elevated neuroticism and perceived stress (35). A cohort study of middle-aged adults demonstrated that high neuroticism increases the risk of asthma (36). Another study also indicates that higher neuroticism scores are linked to a greater likelihood of a lifetime asthma diagnosis (37). Furthermore, a seven-year longitudinal study found that neuroticism was predictive of an increased risk of bronchitis (38). It is worth mentioning that there is substantial evidence that nocturnal hypoxemia frequently occurs in conditions such as asthma (39, 40), COPD (39, 41), OSA (42) and bronchitis (43).

The relationship between neuroticism and nocturnal hypoxia can be understood through both autonomic dysfunction and hormonal regulation. Existing research also highlights that neuroticism is associated with autonomic dysfunction (44, 45), particularly through increased sympathetic activity (46, 47). This heightened sympathetic activity can disrupt normal respiratory patterns, with studies indicating interactions between muscle sympathetic nerve activity and hypoxia (48). Additionally, neuroticism is associated with an imbalance between sympathetic and parasympathetic nervous system activity (49, 50). Increased sympathetic activity, coupled with reduced parasympathetic regulation, can lead to peripheral vasoconstriction, which reduces oxygen delivery to tissues. This imbalance may be especially pronounced during sleep, where reduced airway dilation due to sympathetic influence can result in intermittent breathing pauses and hypoxia (51). Moreover, individuals with high levels of neuroticism are more prone to anxiety and panic reactions, especially in quiet, isolated environments at night. These responses can lead to heightened awareness of breathing patterns and may cause alterations in respiratory function, such as shortness of breath or hyperventilation, which in turn can lead to hypoxemia (52). In addition, the connection between neuroticism and nocturnal hypoxia can also be explained through its effects on the hypothalamic-pituitary-adrenal (HPA) axis. Neuroticism is linked to prolonged HPA axis activation (53–55), which leads to elevated cortisol levels. These hormonal changes affect not only metabolic processes but also the central nervous system’s control of respiration (56, 57). This can result in irregular breathing patterns, increasing the risk of nocturnal hypoxia (58, 59).

While substantial evidence exists on the significant associations between personality traits, nocturnal hypoxia, and psychological symptoms, no prior study has investigated the potential mediating role of nocturnal hypoxia in this intricate relationship. Therefore, our study aims to fill this gap and contribute to the existing literature in several meaningful ways. Our primary objective is to examine the hypothesis that nocturnal hypoxia mediates the positive association between personality traits and psychological symptoms in individuals experiencing sleep disturbances. By addressing this gap in knowledge, our study seeks to advance understanding of the complex interplay between personality traits, nocturnal hypoxia, and psychological symptoms, thereby providing valuable insights for clinical practice and further research in this field.

## 2 Methods

### 2.1 Participants

We recruited a total of 171 participants aged 18 and above, presenting with sleep complaints (44 males, with a mean age of 57.99 years), from the outpatient or inpatient department of Dalian Seventh People’s Hospital between September 2019 and January 2023. All participants underwent polysomnography (PSG) due to various sleep-related issues, such as sleep disorders, snoring, abnormal behavior during sleep, observed apnea, and other related complaints. General information gathered for each participant included gender, age, marital status, family history of mental disorders, smoking and drinking history, major illnesses, medication use, and body mass index, among others. All procedures involving human participants adhered to the ethical standards set forth by the institutional and/or national research committee and complied with the principles outlined in the 1964 Helsinki Declaration and its subsequent amendments or comparable ethical standards. The study received approval from the ethics committee of Dalian Seventh People’s Hospital, and written informed consent was obtained from all participants or their legal guardians.

### 2.2 PSG measures

Nocturnal PSG serves as a crucial diagnostic tool for various sleep disorders, allowing for a comprehensive assessment of sleep architecture, respiratory patterns, and hypoxia levels in subjects. PSG enables simultaneous recording of electroencephalogram (EEG), electromyography (EMG), and eye movement, providing detailed insights into sleep quality and respiratory events (60, 61). In our study, participants underwent PSG using a 64-channel PSG system (Compumedics Grael, Australia), adhering to the guidelines outlined by the American Academy of Sleep Medicine. PSG data were meticulously scored to analyze sleep stages and respiratory events. To evaluate hypoxia levels, peripheral capillary oxygen saturation (SpO_2_) measurements were obtained, including average SpO_2_, lowest SpO_2_, and cumulative time spent with oxygen saturation levels (T95, T85) below various thresholds (e.g., <95%, <85%). These measurements were conducted from 22:00 to 06:00 the following day in a controlled environment characterized by darkness and minimal disturbance.

### 2.3 Mental health status measurements

In our study, we employed the Symptom Checklist-90-Revised (SCL-90-R), specifically utilizing its Chinese version, to assess the mental health status of participants. This self-report questionnaire comprehensively evaluates various dimensions of psychological symptoms, encompassing somatization, obsessive compulsive, interpersonal sensitivity, depression, anxiety, hostility, phobic anxiety, paranoid ideation, and psychoticism. Participants were instructed to rate their behaviors, feelings, and thoughts over the preceding week using a 5-point Likert scale, providing a nuanced understanding of their psychological experiences. To enhance the reliability of our assessments, we carefully managed the timing of mental health evaluations relative to overnight PSG administration. Specifically, we restricted the time interval between mental testing and PSG to three days. This precautionary measure aimed to minimize potential fluctuations in mental health status that could confound our analyses. By ensuring consistency in the timing of assessments, we sought to establish a robust foundation for our findings and enhance the validity of our conclusions.

### 2.4 Personality traits measurements

The Eysenck Personality Questionnaire (EPQ), devised by Hans J. Eysenck, is a self-report scale derived from the Eysenck Personality Inventory (EPI). Rooted in Eysenck’s personality dimension framework, the EPQ evaluates personality traits via factor analysis of questionnaire data. Eysenck identified three core dimensions of personality type through this method: extraversion (E), neuroticism (N), and psychoticism (P). Additionally, the questionnaire incorporates a social desirability scale (L), enhancing its comprehensive assessment of personality traits. Thanks to its brevity, simplicity, and broad applicability, the EPQ has exhibited strong reliability and validity, rendering it a valuable instrument in both psychological research and clinical settings.

### 2.5 Statistical analysis

Statistical analyses were performed using the Statistical Package for the Social Sciences (SPSS Inc., Chicago, IL, USA, version 23.0). Descriptive statistics were computed for variables including gender, age, marital status, family history of mental disorders, smoking history, drinking history, major illness, medication use, and BMI. Absolute and relative frequencies were calculated for qualitative variables. The student’s t-test was employed to compare means between two groups, while analysis of variance (ANOVA) was used to compare the means of more than three groups. To assess for a significant indirect effect, a mediation analysis was conducted using SPSS 25.0, examining three pathways: pathway A from the independent variable to the mediator, pathway B from the mediator to the dependent variable, and pathway C from the independent variable to the dependent variable. Variables demonstrating a p-value < 0.05 in the bivariate analysis were included in the path analysis. Statistical significance was set at p < 0.05.

## 3 Results

### 3.1 Characteristics of participants

The study comprised a total of 171 participants, with 44 being male and having an average age of 58.84 years (standard deviation ±10.11). Among the participants, the majority were married (91.8%), with 1.8% being divorced, 2.9% single, and 3.6% widowed. Noteworthy findings included 12.3% reporting a family history of mental disorders, 22.8% with a history of hypertension, 9.4% with a history of diabetes, 17% with a history of fatty liver, 12.3% with a history of cerebral infarction or lacunar infarction, 10.5% with a smoking history, 7.6% with a history of alcohol consumption, 8.8% with a history of COPD, 4.1% with a history of asthma, and 18.1% with a history of OSA. Furthermore, 70.8% of participants had used at least one benzodiazepine within a week, while 15.8% had used more than one benzodiazepine during the same timeframe (**Table 1**).

**Table 1:** Characteristics of the participants.

Variable	Category	N (%)
Gender	Male	44(25.7%)
	Female	127 (74.3%)
Marital status	Married	157 (91.8%)
	Divorced	3 (1.8%)
	Widow (er)	6 (3.5%)
	Single	5 (2.9%)
Family history of mental disorders	Yes	21 (12.3%)
	No	150 (87.7%)
History of hypertension	Yes	39 (22.8%)
	No	132 (77.2%)
History of diabetes	Yes	16 (9.4%)
	No	155 (90.6%)
History of fatty liver	Yes	29 (17.0%)
	No	142 (83.0%)
History of cerebral infarction or lacunar infarction	Yes	21 (12.3%)
	No	150 (88.7%)
Smoking history	Yes	18 (10.5%)
	No	153 (89.5%)
Drinking history	Yes	13 (7.6%)
	No	158 (92.4%)
Benzodiazepine use	0	23 (13.5%)
	1	121 (70.8%)
	>1	27 (15.8%)
History of COPD	Yes	15 (8.8%)
	No	156 (91.2%)
History of asthma	Yes	7 (4.1%)
	No	164 (95.9%)
History of OSA	Yes	31 (18.1%)
	No	140 (81.9%)
Variable	Mean	SD
Age	57.99	11.27
BMI	23.06	3.01
T95	1.93	2.05
T85	0.17	0.59
EPQ score		
E	52.0	10.96
N	57.92	11.57
P	52.81	12.18
SCL-90-R score		
Somatization	2.09	0.72
Obsessive compulsive	2.30	0.82
Interpersonal sensitivity	1.94	0.74
Depression	2.22	0.83
Anxiety	2.20	0.88
Hostility	1.79	0.72
Phobic anxiety	1.75	0.81
Paranoid ideation	1.69	0.70
Psychoticism	1.81	0.67

SD, standard deviation; COPD, chronic obstructive pulmonary disease; BMI, body mass index; T95%, the cumulative time of oxygen saturation<95%; T85%, the cumulative time of oxygen saturation<85%; E, extraversion; N, neuroticism; P, psychoticism; SCL-90-R, Symptom Checklist-90-R.

In all participants, the mean BMI was 23.06 ± 3.01. The variables T95 and T85 represented the cumulative time of nocturnal hypoxia, with EPQ scores for extraversion (E) averaging 52.10 (± 0.96), neuroticism (N) averaging 57.92 (± 1.57), and psychoticism (P) averaging 52.81 (± 12.18). The SCL-90-R scores reflected the psychological status of the participants across nine dimensions: somatization (2.09 ± 0.72), obsessive compulsive (2.30 ± 0.82), interpersonal sensitivity (1.94 ± 0.74), depression (2.22 ± 0.83), anxiety (2.20 ± 0.88), hostility (1.79 ± 0.72), phobic anxiety (1.75 ± 0.81), paranoid ideation (1.69 ± 0.70), and psychoticism (1.81 ± 0.67) (**Table 1**).

### 3.2 Bivariate analysis

The results of bivariate analysis, presented in **Tables 2** and **3**, revealed significant associations between various factors and psychological symptoms. Participants with a family history of mental disorders exhibited higher scores in somatization (2.34 ± 0.54 vs 2.05 ± 0.73, p < 0.05), obsessive-compulsive (2.52 ± 0.54 vs 2.27 ± 0.85, p < 0.05), and depression (2.57 ± 0.72 vs 2.17 ± 0.84, p < 0.05) compared to those without such a family history. Similarly, participants with a smoking history demonstrated elevated levels of depression, anxiety, hostility, phobic anxiety, paranoid ideation, and psychoticism compared to non-smokers (p < 0.05). Likewise, participants with a history of alcohol consumption displayed increased scores in depression, anxiety, hostility, paranoid ideation, and psychoticism compared to those without a history of alcohol consumption (p < 0.05) (**Table 2**).

**Table 2:** Bivariate analysis of factors associated with psychological symptoms (somatization, obsessive compulsive, interpersonal sensitivity, depression, anxiety, hostility, phobic anxiety, paranoid ideation, psychoticism).

Variable	Category	somatization	obsessive compulsive	interpersonal sensitivity	depression	anxiety	hostility	phobic anxiety	paranoid ideation	psychoticism
		Mean (SD)	Mean (SD)	Mean (SD)	Mean (SD)	Mean (SD)	Mean (SD)	Mean (SD)	Mean (SD)	Mean (SD)
Gender	Male	1.92 (0.68)	2.25 (0.71)	2.00 (0.79)	2.17 (0.76)	2.14 (0.82)	1.84 (0.84)	1.85 (0.92)	1.77 (0.70)	1.88 (0.68)
	Female	2.14 (0.73)	2.31 (0.85)	1.93 (0.73)	2.24 (0.86)	2.22 (0.89)	1.777(0.68)	1.74 (0.78)	1.66 (0.70)	1.79 (0.66)
Marital status	Married	2.07 (0.73)	2.27 (0.79)	1.90 (0.70)	2.19 (0.83)	2.17 (0.87)	1.75 (0.68)	1.73 (0.80)	1.64 (0.67)	1.79 (0.66)
	Divorced	2.16 (0.25)	2.20 (0.53)	2.02 (0.67)	2.28 (0.50)	2.50 (0.53)	1.83 (0.47)	2.30 (0.87)	1.66 (0.15)	1.73 (0.32)
	Widow (er)	2.22 (0.60)	2.50 (1.04)	2.43 (0.96)	2.57 (0.88)	2.45 (0.93)	2.03 (0.74)	1.83 (1.02)	2.07 (0.83)	1.92 (0.64)
	Single	2.44 (0.85)	3.06 (1.41)	2.86 (1.09)	2.80 (0.90)	2.80 (1.16)	2.84 (1.32)	2.42 (0.90)	2.68 (0.99)	2.4 0.90)
Family history of mental disorders	No	2.05 (0.73)*	2.27 (0.85)*	1.91 (0.72)	2.17 (0.84)*	2.17 (0.88)	1.75 (0.68)	1.74 (0.79)	1.65 (0.68)	1.78 (0.66)
	Yes	2.34 (0.54)*	2.52 (0.54)*	2.23 (0.83)	2.57 (0.72)*	2.47 (0.82)	2.10 (0.92)	1.97 (0.95)	1.93 (0.79)	2.04 (0.68)
History of hypertension	No	2.09 (0.71)	2.33 (0.84)	1.97 (0.78)	2.23 (0.85)	2.22 (0.90)	1.82 (0.75)	1.72 (0.81)	1.71 (0.71)	1.83 (0.68)
	Yes	2.10 (0.74)	2.19 (0.73)	1.87 (0.61)	2.18 (0.78)	2.15(0.79)	1.70 (0.61)	1.94 (0.82)	1.62 (0.68)	1.74 (0.62)
History of diabetes	No	2.08 (0.71)	2.30 (0.83)	1.94 (0.74)	2.23 (0.84)	2.20 (0.87)	1.78 (0.70)	1.73 (0.78)	1.68 (0.69)	1.80 (0.66)
	Yes	2.19 (0.83)	2.29 (0.73)	1.97 (0.81)	2.18 (0.81)	2.23 (0.93)	1.89 (0.88)	2.11 (1.08)	1.74 (0.80)	1.90 (0.76)
History of fatty liver	No	2.08 (0.70)	2.32 (0.83)	1.96 (0.75)	2.24 (0.85)	2.22 (0.87)	1.80 (0.71)	1.76 (0.78)	1.67 (0.68)	1.81 (0.67)
	Yes	2.13 (0.80)	2.20 (0.78)	1.88 (0.71)	2.13 (0.73)	2.21 (0.90)	1.74 (0.79)	1.78 (0.97)	1.75 (0.80)	1.80 (0.63)
History of cerebral infarction or lacunar infarction	No	2.06 (0.69)	2.28 (0.81)	1.95 (0.76)	2.21 (0.82)	2.20 (0.89)	1.79 (0.73)	1.76 (0.81)	1.68 (0.71)	1.80 (0.66)
	Yes	2.26 (0.90)	2.47 (0.88)	1.91 (0.64)	2.30 (0.93)	2.20 (081)	1.78 (0.66)	1.80 (0.87)	1.73 (0.67)	1.90 (0.73)
Smoking history	No	2.06 (0.70)	2.26 (0.79)	1.90 (0.71)	2.17 (0.83)*	2.15 (0.86)*	1.72 (0.67)*	1.71 (0.74)*	1.63 (0.65)*	1.76 (0.63)*
	Yes	2.33 (0.86)	2.59 (0.98)	2.30 (0.94)	2.64 (0.76)*	2.63 (0.90)*	2.36 (0.87)*	2.26 (1.20)*	2.17 (0.93)*	2.26 (0.78)*
Drinking history	No	2.07 (0.71)	2.27 (0.81)	1.91 (0.71)	2.19 (0.83)*	2.16 (0.87)*	1.75 (0.69)*	1.73 (0.77)	1.65 (0.68)*	1.78(0.65)*
	Yes	2.26 (0.83)	2.60 (0.90)	2.35(0.96)	2.63 (0.82)*	2.66 (0.83)*	2.25 (0.89)*	2.19 (1.15)	2.13 (0.85)*	2.23 (0.73)*
Benzodiazepine use	0	2.04 (0.67)	2.03 (0.72)	1.73 (0.59)	1.95 (0.71)	2.04 (0.80)	1.74 (0.63)	1.69 (0.72)	1.57 (0.52)	1.61 (0.59)
	1	2.10 (0.74)	2.35 (0.85)	1.95 (0.76)	2.24 (0.86)	2.19 (0.89)	1.78 (0.73)	1.76 (0.82)	1.67 (0.72)	1.83 (0.70)
	>1	2.09 (0.70)	2.32 (2.30)	2.10 (0.75)	2.37 (0.79)	2.39 (0.86)	1.89(0.74)	1.89 (0.87)	1.84 (0.74)	1.93 (0.53)

SD, standard deviation; *p value<0.05; Groups with p<0.05 are shown in bold font.

**Table 3:** Correlation of continuous variables with psychological symptoms (somatization, obsessive compulsive, interpersonal sensitivity, depression, anxiety, hostility, phobic anxiety, paranoid ideation, psychoticism).

	1	2	3	4	5	6	7	8
1. Somatization	1	-0.13	-0.07	-0.012	0.06	0.02	0.27**	0.40**
1. Obsessive compulsive	1	-0.11	-0.07	-0.08	0.08	-0.06	0.38**	0.31**
1. Interpersonal sensitivity	1	-0.13	-0.04	0.06	0.12	-0.04	0.48**	0.44**
1. Depression	1	-0.12	0.01	-0.01	0.15	0.00	0.44**	0.43**
1. Anxiety	1	-0.11	-0.03	-0.04	0.15	0.03	0.39**	0.38**
1. Hostility	1	-0.15	0.06	-0.02	0.13	0.04	0.41**	0.42**
1. Phobic Anxiety	1	-0.05	0.04	0.02	0.16*	-0.03	0.33**	0.34**
1. Paranoid ideation	1	-0.07	0.02	-0.03	0.09	0.01	0.39**	0.47**
1. Psychoticism	1	-0.12	0.04	0.01	0.15	0.02	0.44**	0.49**
2. Age	-0.13	1						
3. BMI	-0.07	0.13	1					
4. T95	-0.012	0.34**	0.26**	1				
5. T85	0.06	0.10	0.27**	0.39**	1			
6. E	0.02	0.07	0.03	-0.03	-0.17*	1		
7. N	0.27**	-0.09	-0.03	0.06	0.02	-0.25**	1	
8. P	0.40**	-0.03	0.07	0.04	0.07	0.08	0.15*	1

BMI, body mass index; T95%, the cumulative time of oxygen saturation<95%; T85%, the cumulative time of oxygen saturation<85%; E, extraversion; N, neuroticism; P, psychoticism; *p value<0.05; **p value<0.01; Variables with p < 0.05 in the bivariate analysis are highlighted in bold.

Furthermore, our analysis revealed significant associations between neuroticism (N) and psychoticism (P) with all examined psychological symptoms, encompassing somatization (N: Rho=0.27, p < 0.01; P: Rho=0.40, p < 0.01), obsessive compulsive (N: Rho=0.38, p < 0.01; P: Rho=0.31, p < 0.01), interpersonal sensitivity (N: Rho=0.48, p < 0.01; P: Rho=0.44, p < 0.01), depression (N: Rho=0.44, p < 0.01; P: Rho=0.43, p < 0.01), anxiety (N: Rho=0.039, p < 0.01; P: Rho=0.38, p < 0.01), hostility (N: Rho=0.41, p < 0.01; P: Rho=0.42, p < 0.01), phobic anxiety (N: Rho=0.33, p < 0.01; P: Rho=0.34, p < 0.01), paranoid ideation (N: Rho=0.39, p < 0.01; P: Rho=0.47, p < 0.01), and psychoticism (N: Rho=0.44, p < 0.01; P: Rho=0.49, p < 0.01). Additionally, the cumulative time of nocturnal hypoxia, denoted by T85, exhibited a significant association with phobic anxiety (Rho=0.16, p < 0.05). Additionally, T95 was found to be associated with both age (Rho=0.34, p < 0.01) and BMI (Rho=0.26, p < 0.01). Moreover, T85 demonstrated associations with both BMI (Rho=0.27, p < 0.01) and extraversion (E) personality traits (Rho=0.39, p < 0.01) (**Table 3**).

### 3.3 Mediation analysis

**Figure 1:**
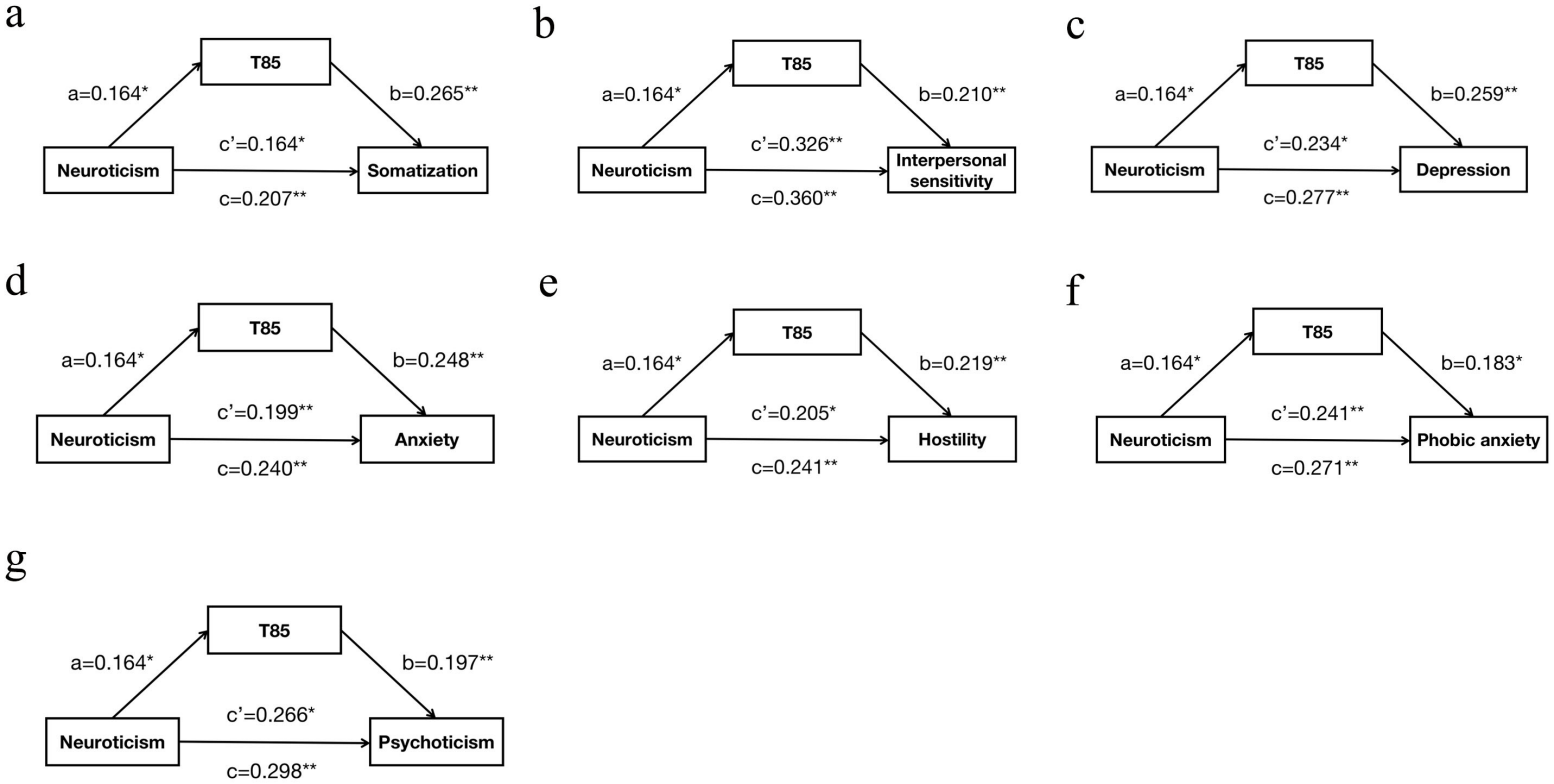
The mediating role of T85 in the relationship between neuroticism and psychological symptoms. **(A)**: T85 as a mediator between neuroticism and somatization; **(B)**: T85 as a mediator between neuroticism and interpersonal sensitivity; **(C)**: T85 as a mediator between neuroticism and depression; **(D)**: T85 as a mediator between neuroticism and anxiety; **(E)**: T85 as a mediator between neuroticism and hostility; **(F)**: T85 as a mediator between neuroticism and phobic anxiety; **(G)**: T85 as a mediator between neuroticism and psychoticism; Pathway: (a) Relation between neuroticism and T85; (b) Relation between T85 and psychological symptoms; (c) Total effect of neuroticism on psychological symptoms; (c’) Direct effect of neuroticism on psychological symptoms. Numbers are displayed as regression coefficients (standard error). *p*<0.05; *p<0.01.

The mediation analysis, adjusted for gender, age, marital status, family history of mental disorders, smoking history, drinking history, and BMI, revealed that T85 played a partial mediating role in the association between neuroticism and various psychological symptoms. Specifically, T85 partially mediated the relationship between neuroticism and somatization (c=0.207, c’=0.164, a*b=0.043, proportion of mediation 20.8%), interpersonal sensitivity (c=0.360, c’=0.326, a*b=0.034, proportion of mediation 9.6%), depression (c=0.277, c’=0.234, a*b=0.042, proportion of mediation 15.3%), anxiety (c=0.240, c’=0.199, a*b=0.041, proportion of mediation 16.9%), hostility (c=0.241, c’=0.205, a*b=0.036, proportion of mediation 14.9%), phobic anxiety (c=0.271, c’=0.241, a*b=0.030, proportion of mediation 11.1%), and psychoticism (c=0.298, c’=0.266, a*b=0.032, proportion of mediation 10.8%). Higher scores of neuroticism were significantly associated with increased levels of T85, which, in turn, were associated with higher scores of these psychological symptoms. Additionally, higher scores of neuroticism were directly associated with elevated scores of these symptoms (**Figure 1**).

## 4 Discussion

Regarding the direct effect, our findings revealed that greater psychological symptoms, including somatization, interpersonal sensitivity, depression, anxiety, hostility, phobic anxiety, and psychoticism, were directly associated with neuroticism. This is consistent with previous research that has also confirmed the link between neuroticism and psychological symptoms (15, 16). Indeed, research has found that neuroticism plays a significant moderating role between allostatic load and mental health during the COVID-19 pandemic (62). This suggests that individuals with higher levels of neuroticism may be more susceptible to the effects of allostatic load on their mental well-being during times of heightened stress and uncertainty, such as a global pandemic. Allostatic load refers to the cumulative physiological burden on the body due to chronic stressors, and individuals with high neuroticism may experience greater difficulty in coping with these stressors, leading to exacerbated mental health outcomes (62). Additionally, other studies have shown that momentary self-regulation is more variable in individuals with higher levels of neuroticism (63). It’s been suggested that neuroticism personality traits can influence physiological reactivity to mental and emotional stressors, potentially increasing psychological stress in various complex situations (64–66). Exposure to pandemic stressors combined with neuroticism has been identified as a risk factor for depressive symptoms, aiding in the identification of individuals at greater risk for adverse psychological responses during the COVID-19 pandemic (67). Some scholars propose that neuroticism serves as an independent etiologically informative risk factor for common mental disorders and can serve as an efficient marker of nonspecific general risk in mental health (68). Moreover, genome-wide association studies have revealed genetic correlations between neuroticism and conditions such as anorexia nervosa, major depressive disorder, and schizophrenia (69). A meta-analysis has further supported associations between neuroticism and anxiety, depression, and non-specific mental distress (65, 70). In conjunction with our findings, these research results collectively underscore the crucial role of neuroticism in understanding mental health outcomes and risk factors for various psychological conditions. Neuroticism emerges as a significant factor influencing how individuals respond to stressors and navigate psychological challenges, particularly during challenging periods like the COVID-19 pandemic.

It is worth mentioning that during the COVID-19 pandemic, the high incidence of hypoxia has further highlighted its role in the development of psychiatric symptoms among neurotic populations. The COVID-19 pandemic has been linked to significant respiratory dysfunctions, particularly in patients with pre-existing conditions. Studies indicate that COVID-19 exacerbates underlying respiratory diseases such as COPD, asthma, and OSA, which are also associated with neuroticism (71–73). In severe cases of COVID-19, the virus can directly impact lung function, often resulting in acute respiratory distress syndrome and subsequent nocturnal hypoxia (74). Additionally, asymptomatic hypoxia is more prevalent in COVID-19 cases and is linked to adverse outcomes and altered mental status (75–77). This is particularly relevant for individuals with neurotic traits, who may be more vulnerable to respiratory complications due to stress-induced physiological changes, including autonomic dysfunction and impaired breathing regulation (35, 78). Moreover, the pandemic has been associated with increased distress, fear, obsessions, and reduced positive mood symptoms. Individuals with neuroticism and autonomic risk factors may be at a heightened risk for developing psychopathology (79). Research has shown that patients with OSA experience approximately an eight-fold greater risk of COVID-19 infection compared to a similar-aged population (80). The relationship between neuroticism and respiratory health during the COVID-19 pandemic suggests that individuals with high neuroticism may face worse outcomes, including increased anxiety and stress, which can further exacerbate respiratory issues such as nocturnal hypoxia. Furthermore, there is evidence that COVID-19-related sleep disturbances and heightened stress can contribute to irregular breathing patterns during sleep, thereby increasing the risk of intermittent nocturnal hypoxia in affected patients (81, 82). In this context, the pandemic underscores the importance of considering both psychological and physiological factors, as the interaction between neuroticism and respiratory health appears especially pertinent during times of heightened stress, such as during COVID-19.

Our mediation analysis confirmed our hypothesis regarding the indirect effect: T85 serves as a mediating variable in the association between neuroticism personality traits and psychological symptoms, including somatization, interpersonal sensitivity, depression, anxiety, hostility, phobic anxiety, and psychoticism. Specifically, a higher cumulative time of nocturnal hypoxia in individuals with neuroticism personality traits was found to be associated with an increased manifestation of psychological symptoms. This study’s mediation analysis elucidated the interplay among these three variables (neuroticism personality traits, T85, and psychological symptoms) and highlighted the mediator role of nocturnal hypoxia in this association, thereby extending the findings of previous literature. Healthcare professionals may potentially identify individuals at greater risk of developing psychological symptoms by identifying those with heightened levels of nocturnal hypoxia. Early identification and intervention targeting nocturnal hypoxia may, therefore, be beneficial in mitigating the development or progression of psychological symptoms in these individuals.

In addition, the accumulation of hypoxia emerges as a crucial mediating factor, indicating that prolonged hypoxia may indirectly contribute to the exacerbation of psychological symptoms. Hypoxia is intricately linked to various diseases, including chronic kidney disease, lung diseases, and anemia. Our experimental findings suggest that, in the presence of a predisposition toward certain personality traits, the cumulative duration of nocturnal hypoxia may serve as a pivotal mediating factor in the transition to mental illness. Furthermore, a big data survey demonstrates that patients with COPD and current smokers are at an increased risk of developing depression (83). Additionally, research has shown that hypobaric hypoxia exposure induces depression-like behavior in female rats, but not in males (84). Previous studies have shown that the total duration of unconsciousness following a hypoxic brain injury is closely associated with long-term cognitive prognosis. In other words, the duration of consciousness impairment is an important predictor of cognitive outcomes. This is consistent with our research findings. These findings underscore the importance of considering nocturnal hypoxia as a potential mediator in the relationship between personality traits and mental illness, highlighting the need for further research to elucidate the underlying mechanisms and implications for clinical practice.

Over the past two decades, insights from clinical practice and animal experiments have illuminated the intricate role of hypoxic injury in the pathophysiology of mental disorders, driven by diverse neurochemical and neurobiological mechanisms. Despite the conventional assumption of a close correlation between neuronal activity and cerebral blood flow (CBF) (85), hypoxia-induced alterations in CBF exhibit regional variability, with specific brain regions affected, leading to neurocognitive dysfunction (86). Previous studies have underscored the detrimental impact of prolonged hypoxia on dopaminergic and serotonergic functions, thereby heightening susceptibility to psychological disorders (87–89). Additionally, hypoxic conditions have been shown to modulate signaling pathways such as brain-derived neurotrophic factor and Ras homolog gene family member A/Ras homolog-associated kinase in the brain (15, 90), culminating in elevated levels of inflammatory mediators and subsequent cerebral hypoxic damage (91–93). Acute hypoxia prompts the deployment of multiple oxygen sensors by neurons to adapt to physiological changes, whereas chronic hypoxia may induce severe perturbations in synaptic transmission and activate transcription factors regulating oxygen homeostasis (94). The brain’s decompensation response to chronic hypoxia may contribute to the emergence of mental symptoms (16), embodying adaptive mechanisms employed by the brain to cope with prolonged oxygen deficiency. Furthermore, primary necrosis initiated by the underlying event triggers a secondary inflammatory process (95). Furthermore, research has revealed that monoamine oxidase A upregulation induced by chronic intermittent hypoxia plays a significant pathogenic role in oxidative stress, inflammation, and cytokine-responsive indoleamine 2,3-dioxygenase-1 activation, resulting in serotonin depletion and neurodegeneration (96). This may be a multifaceted reason why hypoxia plays a role in mediating the relationship between neuroticism and psychological symptoms.

By specifically examining nocturnal hypoxia, our study both confirms and extends earlier findings regarding its significant role in mental health outcomes. These results provide preliminary insights into new opportunities for reducing psychological distress in individuals experiencing hypoxia. Indeed, our findings suggest that prolonged hypoxia during sleep may contribute to neurotic tendencies in personality and predisposition to mental illness. Recognizing the impact of nocturnal hypoxia enables the development of tailored interventions and strategies aimed at promoting mental well-being and mitigating the risk of psychological disorders among patients with neuroticism personality traits. Incorporating nocturnal hypoxia into clinical assessments and treatment plans of patients with neuroticism personality traits empowers healthcare professionals to address the diverse needs of individuals more effectively, ultimately improving mental health outcomes.

While this study has provided valuable clinically relevant evidence, it is important to acknowledge several limitations. Firstly, our study did not investigate the long-term effects of nocturnal hypoxia. Repeated PSG could offer more precise insights, but it may impose financial constraints and decrease patient compliance. Future research should explore alternative detection methods and indicators to better understand the implications of chronic hypoxia over the long term. Secondly, the cross-sectional design of this study precludes establishing causality between variables. Longitudinal studies would be beneficial in elucidating the temporal relationships between nocturnal hypoxia, neuroticism, and psychological symptoms. Thirdly, due to the scope of our study objectives, we limited the questionnaire items to those directly relevant, in order to streamline administration and reduce participant burden. As a result, we did not consider other potential mediators that are already known in the literature. Lastly, future investigations should delve deeper into the interplay between nocturnal hypoxia, neuroticism, and psychological symptoms. Despite these limitations, this study represents an initial exploration of the relationships between nocturnal hypoxia and mental disorders, offering a fresh perspective and evidence for clinical evaluation and treatment approaches.

## 5 Conclusion

Nocturnal hypoxia may mediate the association between neuroticism personality traits and psychological symptoms in individuals with sleep disorders. Recognizing the mediating role of nocturnal hypoxia could aid in developing targeted interventions to enhance the psychological well-being of individuals with sleep disorders. Further research is needed to explore the underlying mechanisms and implications for personalized treatment approaches.

## Data Availability

The raw data supporting the conclusions of this article will be made available by the authors, without undue reservation.

## References

[1] Joshanloo M. Reciprocal relationships between personality traits and psychological well-being. Br J Psychol. (2023) 114:54–69. doi: 10.1111/bjop.v114.1 36088531

[2] Naragon-Gainey K Simms LJ. Three-way interaction of neuroticism, extraversion, and conscientiousness in the internalizing disorders: evidence of disorder specificity in a psychiatric sample. J Res Pers. (2017) 70:16–26. doi: 10.1016/j.jrp.2017.05.003 29158609 PMC5693372

[3] Subica AM Allen JG Frueh BC Elhai JD Fowler JC. Disentangling depression and anxiety in relation to neuroticism, extraversion, suicide, and self-harm among adult psychiatric inpatients with serious mental illness. Br J Clin Psychol. (2016) 55:349–70. doi: 10.1111/bjc.2016.55.issue-4 26714662

[4] Neeleman J Bijl R Ormel J. Neuroticism, a central link between somatic and psychiatric morbidity: path analysis of prospective data. Psychol Med. (2004) 34:521–31. doi: 10.1017/S0033291703001193 15259837

[5] Bui NH Pasalich DS. Insecure attachment, maladaptive personality traits, and the perpetration of in-person and cyber psychological abuse. J Interpers Violence. (2021) 36:2117–39. doi: 10.1177/0886260518760332 29475418

[6] Benzi IMA Preti E Di Pierro R Clarkin JF Madeddu F. Maladaptive personality traits and psychological distress in adolescence: The moderating role of personality functioning. Pers Individ Differences. (2019) 140:33–40. doi: 10.1016/j.paid.2018.06.026

[7] Rossi C Bonanomi A Oasi O. Psychological wellbeing during the COVID-19 pandemic: the influence of personality traits in the italian population. Int J Environ Res Public Health. (2021) 18:5862. doi: 10.3390/ijerph18115862 34072561 PMC8198634

[8] Roshanmehr H Tavakoli R Khaledi M Fathi J Shafiea SM Aberomand M. Evaluating the activity of salivary enzymes as stress biomarkers under psychological stress and their relationship with rumination and personality traits. Biomarkers. (2021) 26:477–82. doi: 10.1080/1354750X.2021.1919762 33951989

[9] Gonzalez-Bueso V Santamaria JJ Oliveras I Fernandez D Montero E Bano M. Internet gaming disorder clustering based on personality traits in adolescents, and its relation with comorbid psychological symptoms. Int J Environ Res Public Health. (2020) 17:1516. doi: 10.3390/ijerph17051516 32111070 PMC7084409

[10] Nikcevic AV Marino C Kolubinski DC Leach D Spada MM. Modelling the contribution of the Big Five personality traits, health anxiety, and COVID-19 psychological distress to generalised anxiety and depressive symptoms during the COVID-19 pandemic. J Affect Disord. (2021) 279:578–84. doi: 10.1016/j.jad.2020.10.053 PMC7598311 33152562

[11] Sutin AR Stephan Y Luchetti M Terracciano A. Self-reported personality traits are prospectively associated with proxy-reported behavioral and psychological symptoms of dementia at the end of life. Int J Geriatr Psychiatry. (2018) 33:489–94. doi: 10.1002/gps.v33.3 PMC5807122 28869657

[12] Mendez Rubio M Antonietti JP Donati A Rossier J von Gunten A. Personality traits and behavioural and psychological symptoms in patients with mild cognitive impairment. Dement Geriatr Cognit Disord. (2013) 35:87–97. doi: 10.1159/000346129 23364170

[13] Greenop KR Almeida OP Hankey GJ van Bockxmeer F Lautenschlager NT. Premorbid personality traits are associated with post-stroke behavioral and psychological symptoms: a three-month follow-up study in Perth, Western Australia. Int Psychogeriatr. (2009) 21:1063–71. doi: 10.1017/S1041610209990457 19586564

[14] Nordfjaern T Bjerkeset O Moylan S Berk M Grawe RW. Clusters of personality traits and psychological symptoms associated with later benzodiazepine prescriptions in the general population: The HUNT Cohort Study. Addict Behav. (2013) 38:2575–80. doi: 10.1016/j.addbeh.2013.06.010 23811061

[15] Li B Xu Y Quan Y Cai Q Le Y Ma T. Inhibition of rhoA/ROCK pathway in the early stage of hypoxia ameliorates depression in mice via protecting myelin sheath. ACS Chem Neurosci. (2020) 11:2705–16. doi: 10.1021/acschemneuro.0c00352 32667781

[16] Burtscher J Niedermeier M Hufner K van den Burg E Kopp M Stoop R. The interplay of hypoxic and mental stress: Implications for anxiety and depressive disorders. Neurosci Biobehav Rev. (2022) 138:104718. doi: 10.1016/j.neubiorev.2022.104718 35661753

[17] Wang F Liu S Zhang Q Ng CH Cui X Zhang D. Prevalence of depression in older nursing home residents in high and low altitude regions: A comparative study. Front Psychiatry. (2021) 12:669234. doi: 10.3389/fpsyt.2021.669234 34239461 PMC8257928

[18] Kious BM Kondo DG Renshaw PF. Living high and feeling low: altitude, suicide, and depression. Harv Rev Psychiatry. (2018) 26:43–56. doi: 10.1097/HRP.0000000000000158 29517615

[19] Wang X Sun H Cui L Wang X Ren C Tong Z. Acute high-altitude hypoxia exposure causes neurological deficits via formaldehyde accumulation. CNS Neurosci Ther. (2022) 28:1183–94. doi: 10.1111/cns.13849 PMC9253739 35582960

[20] Prabakaran S Swatton JE Ryan MM Huffaker SJ Huang JT Griffin JL. Mitochondrial dysfunction in schizophrenia: evidence for compromised brain metabolism and oxidative stress. Mol Psychiatry. (2004) 9:684–97, 43. doi: 10.1038/sj.mp.4001511 15098003

[21] Byrne M Agerbo E Bennedsen B Eaton WW Mortensen PB. Obstetric conditions and risk of first admission with schizophrenia: a Danish national register based study. Schizophr Res. (2007) 97:51–9. doi: 10.1016/j.schres.2007.07.018 17764905

[22] Schmidt-Kastner R van Os J Esquivel G Steinbusch HW Rutten BP. An environmental analysis of genes associated with schizophrenia: hypoxia and vascular factors as interacting elements in the neurodevelopmental model. Mol Psychiatry. (2012) 17:1194–205. doi: 10.1038/mp.2011.183 22290124

[23] Huang X Lu QL Zhu XM Zeng YB Liu Y Hu HY. Histogenous hypoxia and acid retention in schizophrenia: changes in venous blood gas analysis and SOD in acute and stable schizophrenia patients. Front Psychiatry. (2021) 12:792560. doi: 10.3389/fpsyt.2021.792560 34938217 PMC8685331

[24] Maurer J Rebbapragada V Borson S Goldstein R Kunik ME Yohannes AM. Anxiety and depression in COPD: current understanding, unanswered questions, and research needs. Chest. (2008) 134:43S–56S. doi: 10.1378/chest.08-0342 18842932 PMC2849676

[25] Szaulinska K Plywaczewski R Sikorska O Holka-Pokorska J Wierzbicka A Wichniak A. Obstructive sleep apnea in severe mental disorders. Psychiatr Pol. (2015) 49:883–95. doi: 10.12740/PP/32566 26688840

[26] Parkin AJ Miller J Vincent R. Multiple neuropsychological deficits due to anoxic encephalopathy: a case study. Cortex. (1987) 23:655–65. doi: 10.1016/S0010-9452(87)80055-2 3442999

[27] Caine D Watson JD. Neuropsychological and neuropathological sequelae of cerebral anoxia: a critical review. J Int Neuropsychol Soc. (2000) 6:86–99. doi: 10.1017/S1355617700611116 10761372

[28] Raphael JC Elkharrat D Jars-Guincestre MC Chastang C Chasles V Vercken JB. Trial of normobaric and hyperbaric oxygen for acute carbon monoxide intoxication. Lancet. (1989) 2:414–9. doi: 10.1016/S0140-6736(89)90592-8 2569600

[29] Rahmani M Bennani M Benabdeljlil M Aidi S Jiddane M Chkili T. Neuropsychological and magnetic resonance imaging findings in five patients after carbon monoxide poisoning. Rev Neurol (Paris). (2006) 162:1240–7. doi: 10.1016/S0035-3787(06)75137-2 17151516

[30] Hopkins RO Gale SD Johnson SC Anderson CV Bigler ED Blatter DD. Severe anoxia with and without concomitant brain atrophy and neuropsychological impairments. J Int Neuropsychol Soc. (1995) 1:501–9. doi: 10.1017/S135561770000059X 9375235

[31] Greer DM. Mechanisms of injury in hypoxic-ischemic encephalopathy: implications to therapy. Semin Neurol. (2006) 26:373–9. doi: 10.1055/s-2006-948317 16969737

[32] Terracciano A Stephan Y Luchetti M Gonzalez-Rothi R Sutin AR. Personality and lung function in older adults. J Gerontol B Psychol Sci Soc Sci. (2017) 72:913–21. doi: 10.1093/geronb/gbv161 PMC5926981 26786321

[33] Schlecht NF Schwartzman K Bourbeau J. Dyspnea as clinical indicator in patients with chronic obstructive pulmonary disease. Chron Respir Dis. (2005) 2:183–91. doi: 10.1191/1479972305cd079oa 16541601

[34] Topp M Vestbo J Mortensen EL. Personality traits and mental symptoms are associated with impact of chronic obstructive pulmonary disease on patients’ Daily life. COPD. (2016) 13:773–8. doi: 10.3109/15412555.2016.1168793 27089450

[35] Lu Y Ho R Lim TK Kuan WS Goh DY Mahadevan M. Psychiatric comorbidities in Asian adolescent asthma patients and the contributions of neuroticism and perceived stress. J Adolesc Health. (2014) 55:267–75. doi: 10.1016/j.jadohealth.2014.01.007 24630495

[36] Loerbroks A Apfelbacher CJ Thayer JF Debling D Sturmer T. Neuroticism, extraversion, stressful life events and asthma: a cohort study of middle-aged adults. Allergy. (2009) 64:1444–50. doi: 10.1111/j.1398-9995.2009.02019.x 19254292

[37] Najjab A Palka JM Brown ES. Personality traits and risk of lifetime asthma diagnosis. J Psychosom Res. (2020) 131:109961. doi: 10.1016/j.jpsychores.2020.109961 32105866

[38] Kang W Malvaso A. Can the Big Five personality traits predict ever chance and 7-year risk of clinically diagnosed chronic bronchitis in middle-aged and older adults? J Psychosom Res. (2023) 172:111423. doi: 10.1016/j.jpsychores.2023.111423 37406415

[39] Bohadana AB Hannhart B Teculescu DB. Nocturnal worsening of asthma and sleep-disordered breathing. J Asthma. (2002) 39:85–100. doi: 10.1081/JAS-120002190 11990234

[40] Merikanto I Englund A Kronholm E Laatikainen T Peltonen M Vartiainen E. Evening chronotypes have the increased odds for bronchial asthma and nocturnal asthma. Chronobiol Int. (2014) 31:95–101. doi: 10.3109/07420528.2013.826672 24131153

[41] Chaouat A Weitzenblum E Kessler R Charpentier C Ehrhart M Levi-Valensi P. Sleep-related O2 desaturation and daytime pulmonary haemodynamics in COPD patients with mild hypoxaemia. Eur Respir J. (1997) 10:1730–5. doi: 10.1183/09031936.97.10081730 9272911

[42] Young T Peppard PE Gottlieb DJ. Epidemiology of obstructive sleep apnea: a population health perspective. Am J Respir Crit Care Med. (2002) 165:1217–39. doi: 10.1164/rccm.2109080 11991871

[43] Calverley PM Brezinova V Douglas NJ Catterall JR Flenley DC. The effect of oxygenation on sleep quality in chronic bronchitis and emphysema. Am Rev Respir Dis. (1982) 126:206–10. doi: 10.1164/arrd.1982.126.2.206 7103244

[44] Di Simplicio M Costoloni G Western D Hanson B Taggart P Harmer CJ. Decreased heart rate variability during emotion regulation in subjects at risk for psychopathology. Psychol Med. (2012) 42:1775–83. doi: 10.1017/S0033291711002479 22067596

[45] Tateishi K Ohtani N Ohta M. Physiological effects of interactions between female dog owners with neuroticism and their dogs. J Veterinary Behavior. (2014) 9:304–10. doi: 10.1016/j.jveb.2014.08.005

[46] Peters JR Eisenlohr-Moul TA Walsh EC Derefinko KJ. Exploring the pathophysiology of emotion-based impulsivity: The roles of the sympathetic nervous system and hostile reactivity. Psychiatry Res. (2018) 267:368–75. doi: 10.1016/j.psychres.2018.06.013 PMC6309543 29957555

[47] Paine P Kishor J Worthen SF Gregory LJ Aziz Q. Exploring relationships for visceral and somatic pain with autonomic control and personality. Pain. (2009) 144:236–44. doi: 10.1016/j.pain.2009.02.022 19398272

[48] Jouett NP Watenpaugh DE Dunlap ME Smith ML. Interactive effects of hypoxia, hypercapnia and lung volume on sympathetic nerve activity in humans. Exp Physiol. (2015) 100:1018–29. doi: 10.1113/eph.2015.100.issue-9 26132990

[49] Chida Y Hamer M. Chronic psychosocial factors and acute physiological responses to laboratory-induced stress in healthy populations: a quantitative review of 30 years of investigations. Psychol Bull. (2008) 134:829–85. doi: 10.1037/a0013342 18954159

[50] Thayer JF Lane RD. A model of neurovisceral integration in emotion regulation and dysregulation. J Affect Disord. (2000) 61:201–16. doi: 10.1016/S0165-0327(00)00338-4 11163422

[51] Somers VK Dyken ME Clary MP Abboud FM. Sympathetic neural mechanisms in obstructive sleep apnea. J Clin Invest. (1995) 96:1897–904. doi: 10.1172/JCI118235 PMC185826 7560081

[52] Walsh JJUL Royal Holloway. Type A, neuroticism, and physiological functioning. Pers Individ Differences. (1994) 16:959–65. doi: 10.1016/0191-8869(94)90238-0

[53] McEwen BS. Physiology and neurobiology of stress and adaptation: central role of the brain. Physiol Rev. (2007) 87:873–904. doi: 10.1152/physrev.00041.2006 17615391

[54] Puig-Perez S Almela M Pulopulos MM Hidalgo V Salvador A. Are neuroticism and extraversion related to morning cortisol release in healthy older people? Int J Psychophysiol. (2016) 110:243–8. doi: 10.1016/j.ijpsycho.2016.07.497 27425570

[55] Poppelaars ES Klackl J Pletzer B Wilhelm FH Jonas E. Social-evaluative threat: Stress response stages and influences of biological sex and neuroticism. Psychoneuroendocrinology. (2019) 109:104378. doi: 10.1016/j.psyneuen.2019.104378 31382169

[56] Abelson JL Khan S Giardino N. HPA axis, respiration and the airways in stress–a review in search of intersections. Biol Psychol. (2010) 84:57–65. doi: 10.1016/j.biopsycho.2010.01.021 20144683

[57] Castelli MP Pibiri F Carboni G Piras AP. A review of pharmacology of NCS-382, a putative antagonist of gamma-hydroxybutyric acid (GHB) receptor. CNS Drug Rev. (2004) 10:243–60. doi: 10.1111/j.1527-3458.2004.tb00025.x PMC6741708 15492774

[58] Meuret AE Ritz T. Hyperventilation in panic disorder and asthma: empirical evidence and clinical strategies. Int J Psychophysiol. (2010) 78:68–79. doi: 10.1016/j.ijpsycho.2010.05.006 20685222 PMC2937087

[59] Mohammadi H Rezaei M Sharafkhaneh A Khazaie H Ghadami MR. Serum testosterone/cortisol ratio in people with obstructive sleep apnea. J Clin Lab Anal. (2020) 34:e23011. doi: 10.1002/jcla.23011 31549459 PMC6977109

[60] Leong KW Griffiths A Adams AM Massie J. How to interpret polysomnography. Arch Dis Child Educ Pract Ed. (2020) 105:130–5. doi: 10.1136/archdischild-2018-316031 31615846

[61] Jafari B Mohsenin V. Polysomnography. Clin Chest Med. (2010) 31:287–97. doi: 10.1016/j.ccm.2010.02.005 20488287

[62] Gallagher S Sumner R Creaven AM O’Suilleabhain PS Howard S. Allostatic load and mental health during COVID-19: The moderating role of neuroticism. Brain Behav Immun Health. (2021) 16:100311. doi: 10.1016/j.bbih.2021.100311 34514440 PMC8419239

[63] Klein RJ Robinson MD. Neuroticism as mental noise: Evidence from a continuous tracking task. J Pers. (2019) 87:1221–33. doi: 10.1111/jopy.12469 30802956

[64] Jonassaint CR Why YP Bishop GD Tong EM Diong SM Enkelmann HC. The effects of neuroticism and extraversion on cardiovascular reactivity during a mental and an emotional stress task. Int J Psychophysiol. (2009) 74:274–9. doi: 10.1016/j.ijpsycho.2009.09.012 19818369

[65] Poppe C Crombez G Hanoulle I Vogelaers D Petrovic M. Mental quality of life in chronic fatigue is associated with an accommodative coping style and neuroticism: a path analysis. Qual Life Res. (2012) 21:1337–45. doi: 10.1007/s11136-011-0048-8 22038396

[66] Vassend O Czajkowski NO Roysamb E Nielsen CS. The role of neuroticism and pain in dental anxiety: A twin study. Community Dent Oral Epidemiol. (2023) 51:786–93. doi: 10.1111/cdoe.12763 35633060

[67] Morstead T Zheng J Sin NL Rights JD DeLongis A. Pandemic stressors and depressive symptoms: Examining within- and between-person effects of neuroticism. Pers Individ Dif. (2022) 198:111827. doi: 10.1016/j.paid.2022.111827 35945963 PMC9352559

[68] Ormel J Jeronimus BF Kotov R Riese H Bos EH Hankin B. Neuroticism and common mental disorders: meaning and utility of a complex relationship. Clin Psychol Rev. (2013) 33:686–97. doi: 10.1016/j.cpr.2013.04.003 PMC4382368 23702592

[69] Gale CR Hagenaars SP Davies G Hill WD Liewald DC Cullen B. Pleiotropy between neuroticism and physical and mental health: findings from 108 038 men and women in UK Biobank. Transl Psychiatry. (2016) 6:e791. doi: 10.1038/tp.2016.56 27115122 PMC4872414

[70] Jeronimus BF Kotov R Riese H Ormel J. Neuroticism’s prospective association with mental disorders halves after adjustment for baseline symptoms and psychiatric history, but the adjusted association hardly decays with time: a meta-analysis on 59 longitudinal/prospective studies with 443 313 participants. Psychol Med. (2016) 46:2883–906. doi: 10.1017/S0033291716001653 27523506

[71] Alqahtani JS Oyelade T Aldhahir AM Alghamdi SM Almehmadi M Alqahtani AS. Prevalence, Severity and Mortality associated with COPD and Smoking in patients with COVID-19: A Rapid Systematic Review and Meta-Analysis. PloS One. (2020) 15:e0233147. doi: 10.1371/journal.pone.0233147 32392262 PMC7213702

[72] Salles C Mascarenhas Barbosa H. COVID-19 and obstructive sleep apnea. J Clin Sleep Med. (2020) 16:1647. doi: 10.5664/jcsm.8606 32484776 PMC7970613

[73] Iannella G Vicini C Lechien JR Ravaglia C Poletti V di Cesare S. Association between severity of COVID-19 respiratory disease and risk of obstructive sleep apnea. Ear Nose Throat J. (2024) 103:NP10–NP5. doi: 10.1177/01455613211029783 34318690

[74] Anesi GL Jablonski J Harhay MO Atkins JH Bajaj J Baston C. Characteristics, outcomes, and trends of patients with COVID-19-related critical illness at a learning health system in the United States. Ann Intern Med. (2021) 174:613–21. doi: 10.7326/M20-5327 PMC7901669 33460330

[75] Brouqui P Amrane S Million M Cortaredona S Parola P Lagier JC. Asymptomatic hypoxia in COVID-19 is associated with poor outcome. Int J Infect Dis. (2021) 102:233–8. doi: 10.1016/j.ijid.2020.10.067 PMC7604151 33130200

[76] Okuhama A Ishikane M Hotta M Sato L Akiyama Y Morioka S. Clinical and radiological findings of silent hypoxia among COVID-19 patients. J Infect Chemother. (2021) 27:1536–8. doi: 10.1016/j.jiac.2021.07.002 PMC8264520 34294527

[77] Goyal D Inada-Kim M Mansab F Iqbal A McKinstry B Naasan AP. Improving the early identification of COVID-19 pneumonia: a narrative review. BMJ Open Respir Res. (2021) 8:e000911. doi: 10.1136/bmjresp-2021-000911 PMC8573292 34740942

[78] Javelot H Weiner L Hingray C Freire RC Nardi AE. COVID-19 and its psychological consequences: Beware of the respiratory subtype of panic disorder. Respir Physiol Neurobiol. (2020) 282:103530. doi: 10.1016/j.resp.2020.103530 32818605 PMC7431396

[79] Szenczy AK Nelson BD. Neuroticism and respiratory sinus arrhythmia predict increased internalizing symptoms during the COVID-19 pandemic. Pers Individ Dif. (2021) 182:111053. doi: 10.1016/j.paid.2021.111053 34177026 PMC8213985

[80] Maas MB Kim M Malkani RG Abbott SM Zee PC. Obstructive sleep apnea and risk of COVID-19 infection, hospitalization and respiratory failure. Sleep Breath. (2021) 25:1155–7. doi: 10.1007/s11325-020-02203-0 PMC7521948 32989673

[81] Miller MA Cappuccio FP. A systematic review of COVID-19 and obstructive sleep apnoea. Sleep Med Rev. (2021) 55:101382. doi: 10.1016/j.smrv.2020.101382 32980614 PMC7833740

[82] Rizzo D Libman E Baltzan M Fichten C Bailes S. Impact of the COVID-19 pandemic on obstructive sleep apnea: recommendations for symptom management. J Clin Sleep Med. (2021) 17:429–34. doi: 10.5664/jcsm.8922 PMC7927328 33100266

[83] Riblet NB Gottlieb DJ Hoyt JE Watts BV Shiner B. An analysis of the relationship between chronic obstructive pulmonary disease, smoking and depression in an integrated healthcare system. Gen Hosp Psychiatry. (2020) 64:72–9. doi: 10.1016/j.genhosppsych.2020.03.007 32279024

[84] Kanekar S Bogdanova OV Olson PR Sung YH D’Anci KE Renshaw PF. Hypobaric hypoxia induces depression-like behavior in female Sprague-Dawley rats, but not in males. High Alt Med Biol. (2015) 16:52–60. doi: 10.1089/ham.2014.1070 25803141 PMC4376288

[85] Ainslie PN Subudhi AW. Cerebral blood flow at high altitude. High Alt Med Biol. (2014) 15:133–40. doi: 10.1089/ham.2013.1138 24971767

[86] Brownlee NNM Wilson FC Curran DB Lyttle N McCann JP. Neurocognitive outcomes in adults following cerebral hypoxia: A systematic literature review. NeuroRehabilitation. (2020) 47:83–97. doi: 10.3233/NRE-203135 32716324

[87] Wang J Zhou Y Liang Y Liu Z. A large sample survey of tibetan people on the qinghai-tibet plateau: current situation of depression and risk factors. Int J Environ Res Public Health. (2019) 17:289. doi: 10.3390/ijerph17010289 31906177 PMC6981986

[88] Arregui A Hollingsworth Z Penney JB Young AB. Autoradiographic evidence for increased dopamine uptake sites in striatum of hypoxic mice. Neurosci Lett. (1994) 167:195–7. doi: 10.1016/0304-3940(94)91060-X 8177524

[89] Dunlop BW Nemeroff CB. The role of dopamine in the pathophysiology of depression. Arch Gen Psychiatry. (2007) 64:327–37. doi: 10.1001/archpsyc.64.3.327 17339521

[90] Turovskaya MV Gaidin SG Vedunova MV Babaev AA Turovsky EA. BDNF overexpression enhances the preconditioning effect of brief episodes of hypoxia, promoting survival of GABAergic neurons. Neurosci Bull. (2020) 36:733–60. doi: 10.1007/s12264-020-00480-z PMC7340710 32219700

[91] Pham K Parikh K Heinrich EC. Hypoxia and inflammation: insights from high-altitude physiology. Front Physiol. (2021) 12:676782. doi: 10.3389/fphys.2021.676782 34122145 PMC8188852

[92] Park J Jung S Kim SM Park IY Bui NA Hwang GS. Repeated hypoxia exposure induces cognitive dysfunction, brain inflammation, and amyloidbeta/p-Tau accumulation through reduced brain O-GlcNAcylation in zebrafish. J Cereb Blood Flow Metab. (2021) 41:3111–26. doi: 10.1177/0271678X211027381 PMC8756468 34176340

[93] Brew N Nakamura S Hale N Azhan A Davies GI Nitsos I. Dobutamine treatment reduces inflammation in the preterm fetal sheep brain exposed to acute hypoxia. Pediatr Res. (2018) 84:442–50. doi: 10.1038/s41390-018-0045-5 29976968

[94] Mukandala G Tynan R Lanigan S O’Connor JJ. The effects of hypoxia and inflammation on synaptic signaling in the CNS. Brain Sci. (2016) 6:6. doi: 10.3390/brainsci6010006 26901230 PMC4810176

[95] Wolters EC Strekalova T Munter JPJM Kramer BW. Naive BM-derived stem cells (Neuro-Cells) may modify acute and chronic neurodegenerative disorders by modulating macrophage behaviors. Ageing Neur Dis. (2021) 1:3. doi: 10.20517/and.2021.04

[96] Lam CS Li JJ Tipoe GL Youdim MBH Fung ML. Monoamine oxidase A upregulated by chronic intermittent hypoxia activates indoleamine 2,3-dioxygenase and neurodegeneration. PloS One. (2017) 12:e0177940. doi: 10.1371/journal.pone.0177940 28599322 PMC5466431

